# ChaiQi Decoction Alleviates Vascular Endothelial Injury by Downregulating the Inflammatory Response in ApoE-Model Mice

**DOI:** 10.1155/2021/9415819

**Published:** 2021-02-10

**Authors:** Bingran Wang, Jiayan Zhang, Yuhong Lu, Long Peng, Wenling Yuan, Yuqing Zhao, Liping Zhang

**Affiliations:** ^1^Graduate School, Beijing University of Chinese Medicine, Beijing, China; ^2^Department of Gastroenterology, Dongfang Hospital, Beijing University of Chinese Medicine, Beijing, China

## Abstract

Metabolic syndrome (MetS) is a pathological state of metabolic disorders that primarily occur in human proteins, fats, and carbohydrates. It is a complex cluster of core metabolic disorder syndromes including obesity, hyperglycemia, dyslipidemia, and hypertension, and vascular endothelial injury, occurring over time. The currently available treatment options cannot effectively manage MetS. In our previous research, we revealed ChaiQi decoction (CQD) as an effective prescription for improving MetS; however, the specific mechanism remains unclear. Herein, we assessed the efficacy and mechanism of CQD in ApoE gene knockout (ApoE-) mice. Mice were administered with CQD daily for 12 weeks, and the measurement of their body weight was taken monthly. To evaluate the metabolic levels of mice, we determined the fasting blood glucose (FBG), fasting serum insulin (FINS), insulin resistance index (IRI), triglyceride (TG), total cholesterol (TC), high-density lipoprotein cholesterol (HDL-C), and low-density lipoprotein cholesterol (LDL-C) levels. Furthermore, immunohistochemical analysis was adopted to determine the expression of ICAM-1 and VCAM-1 in vascular endothelium, while an optical microscope was adopted to observe the pathological morphology of abdominal aorta in mice. Tumor necrosis factor-*α* (TNF-*α*), interleukin-6 (IL-6), intercellular cell adhesion molecule-1 (ICAM-1), and vascular cell adhesion molecule-1 (VCAM-1) levels were determined using the ELISA method, whereas Western blotting was used to determine nuclear factor- (NF-) *κ*B p65. Of note, intragastric CQD administration ameliorated ApoE-model mice, as evidenced by reduced levels of FBG, FINS, IRI, TG, TC, and LDL-C. Furthermore, CQD alleviated vascular endothelial injury and regularized the structure of the abdominal aorta by downregulating the expressions of proinflammatory cytokines TNF-*α*, IL-6, ICAM-1, VCAM-1, and NF-*κ*B p65. Overall, these findings advocated that CQD ameliorates metabolic levels and vascular endothelial injury in mice by downregulating the inflammatory response and thus may be utilized as a novel MetS therapy.

## 1. Introduction

Metabolic syndrome (MetS) is a cluster of metabolic diseases that transpire together. With the changing lifestyle and westernized diet in recent years, people are increasingly consuming red meat, processed food, refined grain, sugar, and saturated fatty acids. This elevates the incidence of MetS which is estimated to be 25% in the United States, 23% in Europe, and 20–25% in South Asia [1]. Research showed that the risk of cardiovascular disease potentially is advanced by the symptoms of MetS and could become severe when several abnormalities occur concomitantly; this causes major adverse cardiac events (MACE) [2]. Moreover, the probability of cardiovascular diseases and stroke in MetS patients was previously reported to be 2-3 times higher than in non-MetS patients [3]. A prospective analysis conducted to determine the atherosclerosis risk in a community study indicated an 18% greater risk of incident MetS for individuals with the highest western dietary pattern score [4]. MetS symptoms, such as central obesity, dyslipidemia, insulin resistance (IR), and hypertension, are closely associated with vascular endothelial injury, increased intercellular cell adhesion molecule-1(ICAM-1), and vascular cell adhesion molecule-1 (VCAM-1); this promotes inflammation. According to some investigations, reducing inflammation can prevent injury to vascular endothelium [5].

Research on the risk factors associated with metabolic syndrome is constantly on the rise. Several studies have revealed that inflammation is critically important in the occurrence and development of MetS. A study by Hotamisligil first proposed the new medical concept of metabolic inflammation, emphasizing that this low-grade, chronic systemic inflammation mainly arises due to excess nutrients and metabolites. Metabolic inflammation may have similar signaling pathways with typical inflammation [6]. Under normal circumstances, the body is at a steady-state level, and a dynamic balance is maintained between inflammation and metabolism. However, in case of a metabolic disorder, an imbalance occurs in the immune system, activating inflammatory signaling pathways, and prompts the body to release inflammatory factors or amplify an inflammatory response; this causes IR and eventually results in the occurrence of MetS.

Tumor necrosis factor-*α* (TNF-*α*) plays a vital role in the formation of IR [7]. A specific correlation between TNF-*α* level and hypertension exists. Furthermore, interleukin-6 (IL-6) both participates in inflammation and greatly influences the blood vessels; some findings suggest an important role for IL-6 in limiting endothelial dysfunction in response to angiotensin II [8]. Previous studies have reported that IL-6 is associated with obesity-related IR, which alters the formation of normal subcutaneous adipose tissue and interferes with lipid metabolism [9, 10]. Excess IL-6 levels can affect insulin receptor signal transduction and further induce IR. Besides, nuclear factor- (NF-) *κ*B can mediate inflammation and participate in immune regulation and is also related to IR, central obesity, hypertension, lipid metabolism disorders, glucose metabolism disorders, and vascular remodeling. Increased levels and activation of the above inflammatory factors promote the occurrence of MetS.

At present, no effective treatment for MetS is clinically available, and the existing treatment options eliminate various metabolic abnormalities. Commonly used drugs include insulin sensitizers, lipid-lowering drugs, hypoglycemic drugs, and antihypertensive drugs which purposely control obesity and IR and reduce the occurrence of MACE and diabetes [11]. However, these treatments may cause adverse reactions, among them, cough, elevated serum creatinine, headache, gastrointestinal symptoms, and rashes. Notably, there is a lack of treatment for overall metabolic abnormalities; therefore, novel and effective drugs for MetS treatment should urgently be developed [12, 13].

ChaiQi decoction (CQD) is a new and effective traditional Chinese medicine prescription formulated by Zhang, a professor at the Beijing University of Chinese Medicine. In our previous study, we revealed that CQD relieved IR in mice models of MetS induced by an improper diet [14]. Besides, the diet-induced MetS mice model is unstable, the ApoE-mice are more stable and can highly simulate systemic metabolic and functional abnormality, though the actual mechanism of action is yet to be elucidated. Therefore, the present study sought to comprehensively explore the underlying molecular mechanism by which CQD exerts protective effects in ApoE-mice models.

## 2. Materials and Methods

### 2.1. Preparing CQD and LC-MS Analysis of Identified Compounds

CQD granules were purchased from the Pharmacy Department of Dongfang Hospital, Beijing University of Chinese Medicine (Beijing, China). The CQD granules contained ChaiQi ingredients in equal amounts as follows: Chaihu (radix bupleuri), 10 g; Huangqi (radix astragali), 30 g; Baizhu (rhizoma atractylodis macrocephalae), 10 g; Zhishi (fructus aurantii immaturus), 10 g and Sanqi (pseudoginseng), 3 g. CQD was decocted with boiling water at 100°C for 30 min and was prepared into a solution of 100 mg/ml concentration. Liquid chromatography-mass spectrometry (LC-MS) was used to identify the main components of CQD. Chromatographic separation was carried on a Symmety C18 column and the column temperature was maintained at 25°C. The mobile phase was composed of 0.1% formic acid (A) and acetonitrile (B) with gradient elution system (0–30 min, 90%A; 30–60 min, 90%⟶70%A; 60–100 min, 70%⟶40%A; 100–120 min, 40%⟶10%A) at a flow rate of 0.2 mL/min, and the injection volume was 10 *μ*L. The electrospray ion source (ESI) was used for full scan in both positive and negative modes, 50 psi of nebulizer pressure, 10 L/min of spray gas flow, 350°C of spray gas temperature, 1.00 V of crushing voltage, and 50–1000 m/z of scanning range.

### 2.2. Experimental Animals

All the experimental procedures were approved by the Animal Ethics Committee of Beijing University of Chinese Medicine, following guidelines issued by Regulations of Beijing Laboratory Animal Management. In total, 8 male ApoE-mice as the model group and 8 male ApoE-mice as the CQD group, and 8 male C57BL/6J mice as the blank control group (all mice weighing 20–30 g and 6-week-old; Vital River Laboratory Animal Technology, Beijing, China) were housed in a specific pathogen-free room with a temperature range between 20 and 24ºC, 50–60% humidity, light-controlled environment (12/12 h light/dark cycle), and free access to food and sterile tap water. All animals were allowed to adapt to the living conditions for 7 days before commencing the experiments.

### 2.3. Model Assessment and Experimental Procedure

The MetS model is spontaneously constructed using ApoE-mice. The successful development of the model is judged by detecting the degree of insulin resistance and blood lipid metabolism. CQD and distilled water were administered once daily. The mice in the CQD (8.19 g/kg/day, p.o., *n* = 8) group were administered with CQD for 12 weeks, whereas for the control (*n* = 8) group and model (*n* = 8) group, mice received only distilled water. All mice were sacrificed through cervical dislocation and blood samples collected from the Infraorbital venous plexus. The abdominal aorta was removed and cut into 2 sections. One section was fixed in 4% polyformaldehyde for histological analysis, while the section was stored in the freezer at −80ºC for further analysis.

### 2.4. Analysis of Blood Glucose and Insulin

A measure of the bodyweight of all mice was taken at the same time every month. Using the collected blood samples, fasting blood glucose (FBG) serum levels were assessed using mice ELISA kits (KH Bio-engineering, Shanghai, China), whereas fasting serum insulin (FINS) levels were assessed using mice radioimmunoassay kits (North Institute of Biotechnology, Beijing, China). The insulin resistance index (IRI) was calculated using the homeostasis model HOMA with the formula, IRI = (FBG × FINS)/22.5.

### 2.5. Assay for Serum Lipid Levels

Using the collected blood samples, triglyceride (TG), total cholesterol (TC), high-density lipoprotein cholesterol (HDL-C), and low-density lipoprotein cholesterol (LDL-C) levels were determined using mice ELISA kits (KH Bio-engineering, Shanghai, China).

### 2.6. Histological Analysis

After tissues were fixed in 4% polyformaldehyde for 24 h, they were taken out from polyformaldehyde and washed with running water. Then, tissues were dehydrated using a gradient-force dehydrator, embedded in paraffin blocks, and sectioned to 4 *µ*m a thickness. Thereafter, the sections were stained with hematoxylin and eosin (HE). An optical microscope was used to observe the pathological changes in the aortas.

### 2.7. Immunohistochemical Analysis to Detect ICAM-1 and VCAM-1 Expression in Abdominal Aortic Endothelium

Vessel tissue sections were stained using labeled streptavidin-biotin technique and developed with diaminobenzidine to evaluate the expression levels of ICAM-1 and VCAM-1. Observing the stained area, the brownish-yellow particles in the nucleus or cytoplasm were positive expressions that were darker than the background. We observed 15 different fields of view under each specimen at high magnification and analyzed the data after image acquisition.

### 2.8. Detecting Related Inflammatory Factor

TNF-*α*, IL-6, ICAM-1, and VCAM-1 levels were assessed using mice ELISA kits (R&D Systems, USA), whereas Western blot was used to determine the expression of NF-*κ*B p65, with the protocol as previously described [15]. The concentrations of proteins isolated from aorta abdominal tissues were determined using bicinchoninic acid (BCA) assay (CW-Biotech, Beijing, China). Then, the proteins were separated in 10% SDS-PAGE for 1.5 h before they were transferred to polyvinylidene fluoride (PVDF) membranes. The membranes were probed with NF-*κ*B p65 (1 : 500) and *β*-actin (1 : 1000) antibodies (TDY-Biotech, Beijing, China) and incubated with goat polyclonal secondary antibody to rabbit antibodies (ZSGB-Biotech, Beijing, China). Eventually, densitometry was executed to quantify protein band intensities using the Image-pro analyzer.

### 2.9. Statistical Analysis

All data were expressed as mean ± standard deviation (SD) values. SPSS v22.0 (IBM Corp., Armonk, NY, USA) was used for statistical analyses. A comparison of data between groups was performed using one-way analysis of variance (ANOVA) and nonparametric test. *P* < 0.05 was considered statistically significant.

## 3. Results

### 3.1. Effect of CQD on Body Weight in Mice

**Figure 1:**
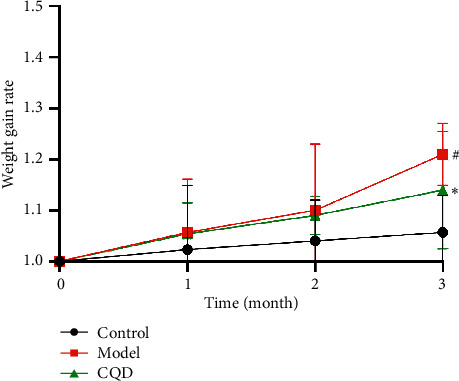
Changes in body weight of mice in each group. Weight gain rate = weight measured in each month/initial weight. Control: blank control group; model: model group; CQD: ChaiQi decoction group. Data are presented as the mean ± SD. ^#^*P* < 0.05 versus the control group; ^*∗*^*P* < 0.05 versus the model group (*n* = 8 mice per group).

All mice's initial weight was 20–30 g, and there was no statistically significant difference in body weight between the groups; all mice adapted effectively to entire experimental conditions, and no deaths occurred. By the end of the 12th week, the weight of mice in each group was higher compared to the initial weight, showing a statistically significant difference, *P* < 0.05. The weight change in the model group was significantly higher compared to the blank control group (1.21 ± 0.061 versus 1.057 ± 0.072, *P* < 0.05). Moreover, the weight change in the CQD group was visibly lower compared to the model group (1.14 ± 0.115 versus 1.21 ± 0.061, *P* < 0.05) (Figure 1).

### 3.2. Identification of Eight Compounds in CQD by LC-MS Analysis

**Figure 2:**
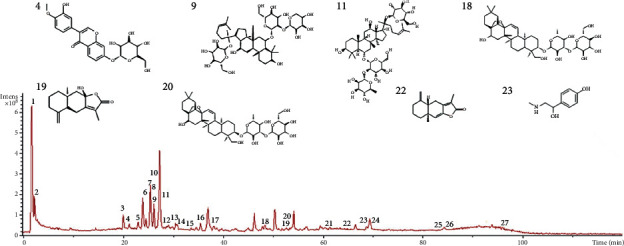
The HPLC fingerprints of BSHXDS components: (4) calycosin-7-glucoside; (9) notoginsenoside R1; (11) ginsenoside Re; (18) saikosaponin A; (19) atractylenolide III; (20) saikosaponin D; (22) atractylenolide I; (23) synephrine.

By contrasting the different chromatographic peaks of CQD and Chinese herbal medicine monomers, we identified 8 compounds from 27 (Figure 2). Eight drug prototype compounds are as shown in Table 1.

### 3.3. Regulatory Effect of CQD on Blood Glucose and Insulin in Mice

**Figure 3:**
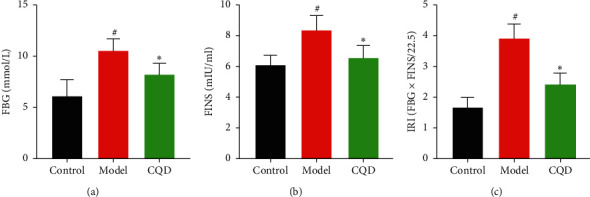
Regulatory effect of CQD on blood sugar and insulin. (a) FBG level; (b) FINS level; (c) IRI level; control: blank control group; model: model group; CQD: ChaiQi decoction group. ^#^*P* < 0.05 versus the control group; ^*∗*^*P* < 0.05 versus the model group (*n* = 8 mice per group).

The FBG level in the model group was significantly higher compared to the blank control group (*P* < 0.05), whereas the level in the CQD group was remarkably lower than that in the model group (*P* < 0.05) (Figure 3(a)). Besides, the FINS level in the model group was higher compared to that in the blank control group (*P* < 0.05), whereas the level in the CQD group was significantly lower than in the model group (*P* < 0.05) (Figure 3(b)). Furthermore, the IRI level in the model group was higher compared to the blank control group (*P* < 0.05), while the level in CQD group was notably lower compared to that in the model group (*P* < 0.05) (Figure 3(c)).

### 3.4. Effect of CQD on Serum Lipid in Mice

**Figure 4:**
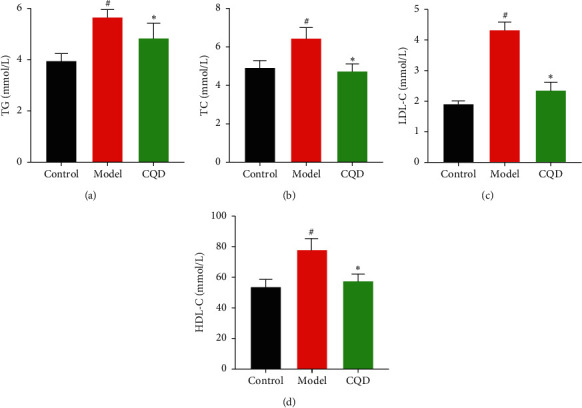
Effect of CQD on serum lipid in mice. (a) TG level; (b) TC level; (c) LDL-C level; (d) HDL-C level. Control: blank control group; model: model group; CQD: ChaiQi decoction group. ^#^*P* < 0.05 versus the control group; ^*∗*^*P* < 0.05 versus the model group (*n* = 8 mice per group).

Here, the TG level in the model group was significantly higher compared to that in the blank control group (*P* < 0.05), while the level in the CQD group was remarkably lower than that in the model group (*P* < 0.05) (Figure 4(a)). Moreover, the TC level in the model group was higher compared to that in the blank control group (*P* < 0.05), whereas the level in the CQD group was significantly lower than that in the model group (*P* < 0.05) (Figure 4(b)). The LDL-C level in the model group was significantly higher compared to that in the blank control group (*P* < 0.05), whereas the level in the CQD group was remarkably lower than that in the model group (*P* < 0.05) (Figure 4(c)). Additionally, the HDL-C level in the model group was higher than that in the blank control and CQD groups (*P* < 0.05) (Figure 4(d)); notably, the increase of HDL-C could be attributed to inflammation.

### 3.5. CQD Improved Histopathology in Mice

**Figure 5:**
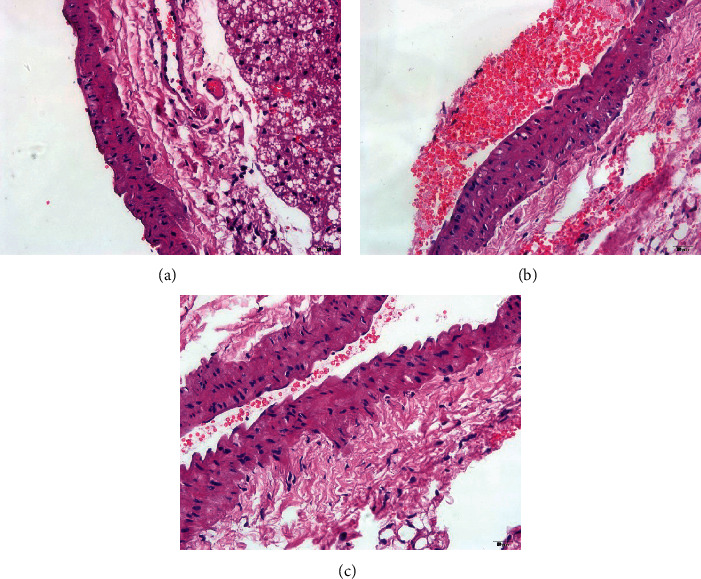
CQD improved histopathology in mice (×400). (a) Blank control group; (b) model group; (c) ChaiQi decoction group.

Compared with the blank control group (Figure 5(a)), the model group showed a damaged structure of the three abdominal aortic walls. The tunica intima was rough and became thick and protruded, and a few endothelial cells were damaged. The smooth muscles of tunica media were uneven showing disordered cells (Figure 5(b)). The manifestation of the CQD group was better than that of the model group (Figure 5(c)): less endothelial cells were damaged. The texture and thickening of the tunica intima, as well as the unevenness of the tunica media, were improved. Also, the smooth muscle cells exhibited a more regular arrangement.

### 3.6. CQD Lowered ICAM-1 and VCAM-1 Expression in Abdominal Aortic Endothelium

**Figure 6:**
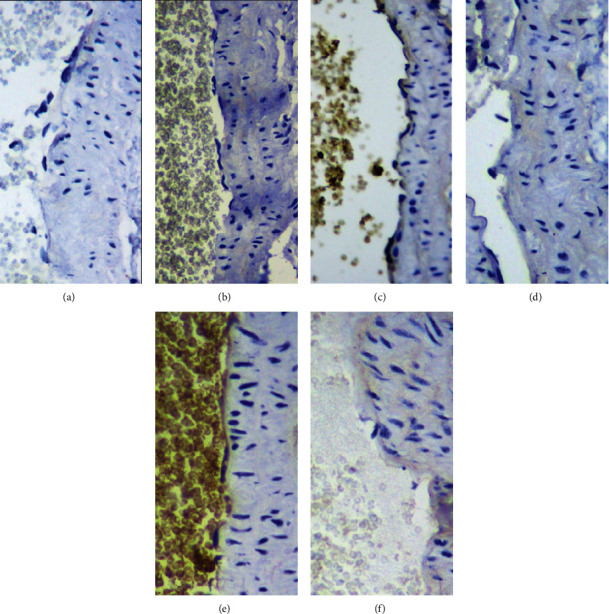
CQD lowered ICAM-1 and VCAM-1 expression in abdominal aortic endothelium. (a) ICAM-1 expression in the blank control group; (b) ICAM-1 expression in the model group; (c) ICAM-1 expression in the ChaiQi decoction group; (d) VCAM-1 expression in the blank control group; (e) VCAM-1 expression in the model group; (f) VCAM-1 expression in ChaiQi decoction group.

Through immunohistochemistry analysis, the brownish-yellow showed positive expression. The expression of ICAM-1 in the blank control group (Figure 6(a)) was extremely low, while, in the model group (Figure 6(b)), brown-yellow particles were highly deposited in the inner membrane, and some were fused into pieces but were significantly more than that in the blank control group. Although the CQD group (Figure 6(c)) showed brown-yellow particles in the inner membrane, the expression level was significantly lower than that in the model group. Similarly, for ICAM-1, the expression of VCAM-1 in the blank control group (Figure 6(d)) was extremely low, whereas it was highly expressed in the inner membrane in the model group (Figure 6(e)); this was significantly higher compared to that in the blank control group. Meanwhile, in the CQD group, the expression of VCAM-1 in the inner membrane was lower compared to the model group (Figure 6(f)).

### 3.7. CQD Inhibited the Expression of TNF-*α*, IL-6, ICAM-1, and VCAM-1 in the Serum of Mice

**Figure 7:**
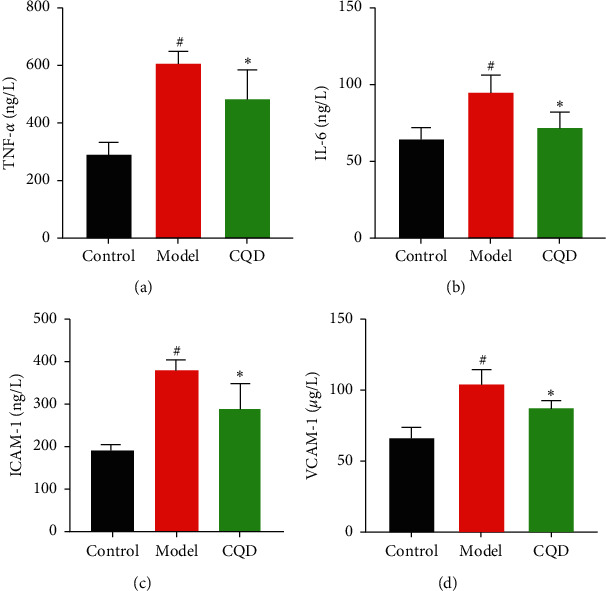
CQD inhibited the expression of TNF-*α*, IL-6, ICAM-1, and VCAM-1 in the serum of mice. Control: blank control group; model: model group; CQD: ChaiQi decoction group. ^#^*P* < 0.05 versus the control group; ^*∗*^*P* < 0.05 versus the model group (*n* = 8 mice per group).

The serum TNF-*α*, IL-6, ICAM-1, and VCAM-1 levels in the model group were significantly higher compared to that in the blank control group (*P* < 0.05). At the same time, TNF-*α*, IL-6, ICAM-1, and VCAM-1 levels in the CQD group were remarkably lower than in the model group (*P* < 0.05) (Figure 7(a)–7(d)).

### 3.8. CQD Lowered the Expression of NF-*κ*B p65 in Mice

**Figure 8:**
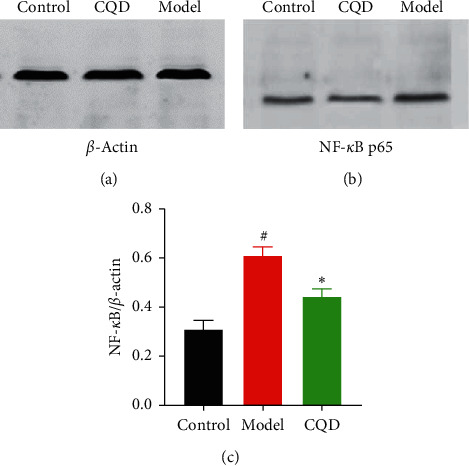
CQD lowered the expression of NF-*κ*B p65 in mice. Control: blank control group; model: model group; CQD: ChaiQi decoction group. ^#^*P* < 0.05 versus the control group; ^*∗*^*P* < 0.05 versus the model group (*n* = 8 mice per group).

The expression of NF-*κ*B p65 in the model group was significantly higher compared to that in the blank control group (*P* < 0.05), whereas NF-*κ*B p65 expression in the CQD group was significantly lower than that in the model group (*P* < 0.05) (Figure 8(a)–8(c)).

## 4. Discussion

Based on the current understanding, MetS is a cluster of metabolic disorders, including central obesity, insulin resistance, diabetes, dyslipidemia, and hypertension. Notably, IR is the core in the progress of MetS and IR can change the metabolism of blood glucose and blood lipids, increase the level of free fatty acids, promote inflammation, accelerate oxidative stress, and alter the spectrum of adipocytokines [16, 17]. Insulin functions promote a reduction in glucose utilization; this elevates blood glucose levels and impairs glucose tolerance. Besides, IR is highly prevalent in MetS patients with vascular endothelial injuries. It can retard endothelium-dependent vasodilation thereby affecting the stability of blood vessels and mediate the formation of atherosclerosis [18]. Similarly, IR is, in most cases, accompanied by hyperinsulinemia. Normal insulin levels are crucial in maintaining vascular endothelial function. However, hyperinsulinemia has been revealed to potentially result in vascular endothelial dysfunction [19]. Normal endothelial cells regulate vascular smooth muscle cells by releasing endothelium-derived factors to maintain vascular tension. Further, it secretes numerous vasoactive substances including nitric oxide (NO) and endothelial contractile factor (ET) which finetune normal vessel integrity and tension stimulated by physiological doses of insulin [20]. Besides, NO is regarded as one of the important indicators of endothelial relaxation function. When Mets develops, the endothelial cells are damaged and NO synthesis is reduced, resulting in reduced endothelium-dependent vasodilation induced by vasodilators such as reactive hyperemia and acetylcholine and high ET secretion [21]. These events cause vasodilation disorders and endothelial dysfunction.

Herein, to reveal the effect of CQD on vascular endothelial injury caused by MetS, we selected ApoE-mice as the model for spontaneous Mets formation. Based on the previous findings, IR is considered to be a key factor in the occurrence and progression of MetS [22]. Therefore, we assessed the formation of MetS by determining the IRI of mice. In our study, the weight of mice increased as the experiment progressed, the disorders of blood glucose and lipid metabolism and IR occurred, indicating that we successfully established the MetS model. Accordingly, in the histological analysis, the abdominal aorta endothelium of the model group was severely damaged, and adhesion molecules were highly accumulated. On the other hand, the IRI and lipid metabolism in mice from the CQD group were lower, suggesting that the CQD had prominent therapeutic effects on ApoE-mice which resulted in MetS and vascular endothelial injury.

Studies have described MetS as a state of systemic low inflammation, and this low-level inflammatory reaction may bridge the components of metabolic syndrome, manifested as an abnormal production of inflammatory factors and activation of inflammatory signaling pathways [23–26]. When IR occurs, TNF-*α* is overexpressed in blood and adipose tissue and is negatively correlated with insulin sensitivity [27]. Many literature reports have confirmed that the high expression of IL-6 during IR is related to the activation of the NF-*κ*B signaling pathway, and after the regulation of body mass index and fat content, the plasma IL-6 level is still associated with insulin sensitivity [28–31]. Moreover, when endothelial dysfunction occurs in the IR state, the expression of ICAM-1 and VCAM-1 is promoted; thus monocytes adhere to endothelial cells in circulation. Then, monocytes and lymphocytes gather under the intima, triggering a series of inflammatory and immune reactions. Other assessments have shown that the NF-*κ*B signaling pathway can be activated in different cells during the IR process. Activation of the NF-*κ*B signaling pathway has been revealed to be closely associated with the occurrence of IR [32].

Vascular injury is a pathological process accompanied by a chronic inflammatory reaction. Inflammatory mediators have been reported to play an important role in the formation and development of vascular injury [33, 34]. When an inflammatory reaction occurs in blood vessels, B lymphocytes, T lymphocytes, and monocyte macrophages are activated, producing numerous inflammatory factors such as IL-6 and TNF-*α*. In our study, under physiological conditions, ICAM-1 and VCAM-1 showed low expression or no expression. Notably, when the blood vessels were injured, they could induce the release of TNF-*α*, rapidly upregulate ICAM-1 and VCAM-1, and enhance the adhesion between leukocytes and vascular endothelial cells, thus activating endothelial cells. Moreover, ICAM-1 and VCAM-1 can promote aggregation of inflammatory cells and release of inflammatory mediators, damage the barrier function of the endothelium, and increase the permeability of vascular wall, thus causing endothelial injury and vascular dysfunction [35]. NF-*κ*B, a ubiquitous factor in tissues and cells, can induce nuclear transcription activation, participate in immune regulation, and mediate an inflammatory response. NF-*κ*B signaling pathway can activate a variety of immune and inflammatory cytokines, for example, TNF-*α*, IL-1, IL-6, ICAM-1, and VCAM-1. The proinflammatory pathway regulated by NF-*κ*B is closely linked to metabolic vascular diseases. Some scholars believe that NF-*κ*B is one of the initiating factors for atherosclerosis [36].

In this study, after observing a definite effect of CQD in ApoE-mice with MetS, we further explored the therapeutic mechanism. We assessed the serum levels of TNF-*α*, IL-1, IL-6, ICAM-1, and VCAM-1 to explore the role of inflammatory factors in the development of MetS and IR and then determined the content of NF-*κ*B using Western blotting. Of note, we revealed that CQD could improve the level of MetS and reduce the injury of vascular endothelium in ApoE-model mice by reducing inflammatory factors and inhibiting the expression of NF-*κ*B related pathway.

In the present study, using ApoE-mice model with vascular endothelial injury, we uncovered that CQD significantly improved the body weight, IRI, and serum lipid level as well as TNF-*α*, IL-6, ICAM-1, VCAM-1, and NF-*κ*B content. The protective effects of CQD may be attributed to its significant regulation of inflammatory response and the NF-*κ*B signaling pathway. Consequently, this reduces the generation and recruitment of inflammatory cytokines and promotes repair of the injured endothelium.

## 5. Conclusion

In this work, we identified eight compounds in CQD by liquid chromatography-mass spectrometry analysis and demonstrated that intragastric administration of CQD has a therapeutic effect on Mets, with IR and serum lipid levels as the main monitoring indicators. Notably, CQD can alleviate MetS and the resulting vascular endothelial injury, and the mechanism may be attributed to a significant downregulation of the inflammatory factors and NF-*κ*B related signaling pathway. These findings suggest that CQD may be utilized as a novel therapeutic option for MetS and the resulting vascular endothelial injury.

## Figures and Tables

**Table 1:** Eight compounds identified in CQD.

No.	Compound	Retention time (min)	Source
4	Calycosin-7-glucoside	20.1	Huangqi (radix astragali)
9	Notoginsenoside R1	26.2	Sanqi (pseudoginseng)
11	Ginsenoside Re	27.3	Sanqi (pseudoginseng)
18	Saikosaponin A	48.3	Chaihu (radix bupleuri)
19	Atractylenolide III	52.6	Baizhu (rhizoma atractylodis macrocephalae)
20	Saikosaponin D	53.4	Chaihu (radix bupleuri)
22	Atractylenolide I	64.8	Baizhu (rhizoma atractylodis macrocephalae)
23	Synephrine	69.4	Zhishi (fructus aurantii immaturus)

## Data Availability

All data are available upon reasonable request from Bingran Wang, bingranwang93@bucm.edu.cn.
